# A new species of *Haploperla* from China (Plecoptera, Chloroperlidae)

**DOI:** 10.3897/zookeys.572.6270

**Published:** 2016-03-15

**Authors:** Zhi-Teng Chen, Yu-Zhou Du

**Affiliations:** 1 School of Horticulture and Plant Protection & Institute of Applied Entomology, Yangzhou University, Yangzhou 225009, China

**Keywords:** Stoneflies, species description, aquatic diversity

## Abstract

A new species of the genus *Haploperla*, *Haploperla triangulata* **sp. n.**, is described and illustrated from specimens collected in Qinghai province, China. *Haploperla triangulata* is characterized by the epiproct mostly sclerotized and hairless, sub-triangular in dorsal view and blunt at tip; and by the aedeagus with two median elliptical lobes ventrally. The new species is compared with its most similar congeners, and diagnostic characters are presented.

## Introduction

The genus *Haploperla* Navás 1934 is mainly distributed in eastern Palaearctic and Nearctic regions, with only thirteen extant species reported (DeWalt et al. 2015). Only four Chinese species of *Haploperla* have been recorded from China, *Haploperla ussurica* Navás, 1934; *Haploperla lepnevae* Zhiltzova & Zwick, 1971; *Haploperla valentinae* Stark & Sivec, 2009; and *Haploperla choui* Li & Yao, 2013 (Navás 1934, Zhiltzova and Zwick 1971, Stark and Sivec 2009, Li et al. 2013). Unknown specimens of *Haploperla* from Shuixia, Huangzhong county in Qinghai province were collected in August 2015, and identified as a new species. The types are deposited in the Insect Collection of Yangzhou University, Jiangsu. The species described in this contribution increases the number of known Chinese *Haploperla* taxa to five.

## Materials and methods

All type specimens are preserved in 75% or 100% ethanol. Specimens were examined and illustrated using a Leica stereomicroscope-MZAPO. Images were taken using a Leica SZ45. The holotype of the new species is deposited in the Insect Collection of Yangzhou University, China.

## Results

### Haploperla triangulata sp. n.

Taxon classificationAnimaliaPlecopteraChloroperlidae

http://zoobank.org/F07E92CF-06D6-418C-B7E9-E840202F3DB6

Figs 1–4 , 5–8 , 9–10

#### Type material.

Holotype. 1 male, China: Qinghai province, Huangzhong county, Shuixia, 101°41.25'E, 36°82.46'N, 2590 m, 8 August, 2015, leg. Yu-Zhou Du. Paratype: 1 male and 7 females, the same locality and data as holotype, leg. Yu-Zhou Du, Zhi-Hou Li, Qiu-Yu Fan.

#### Diagnosis.

This species is characterized by a pale head, a pale pronotum disc with median stripe and brown margins, and the abdominal terga 1–8 with a longitudinal stripe. Epiproct mostly sclerotized and hairless, sub-triangular in dorsal view with a blunt tip. Aedeagus ventrally with two median situated elliptical lobes.

This new species is most similar to *Haploperla valentinae* Stark & Sivec, 2009, known from Sichuan Province, China, but differs in the formation of the epiproct, which is mostly sclerotized and hairless in *Haploperla triangulata* sp. n., whereas it is with sclerotized margins, membranous posterodorsal area and sparse patch of setae in *Haploperla valentinae* (see Figs 13 and 17 in Stark and Sivec 2009). Besides, the subgenital plate of female is originating from tergum 8 to the posterior margin of tergum 9 in *Haploperla triangulata*, while it’s slightly produced as a small rounded lobe with long setae in *Haploperla valentinae* (see fig. 15 in Stark and Sivec 2009). The details of the wing venation and aedeagus are not described for *Haploperla valentinae*.

#### Adult habitus.

**Figures 1–4.**
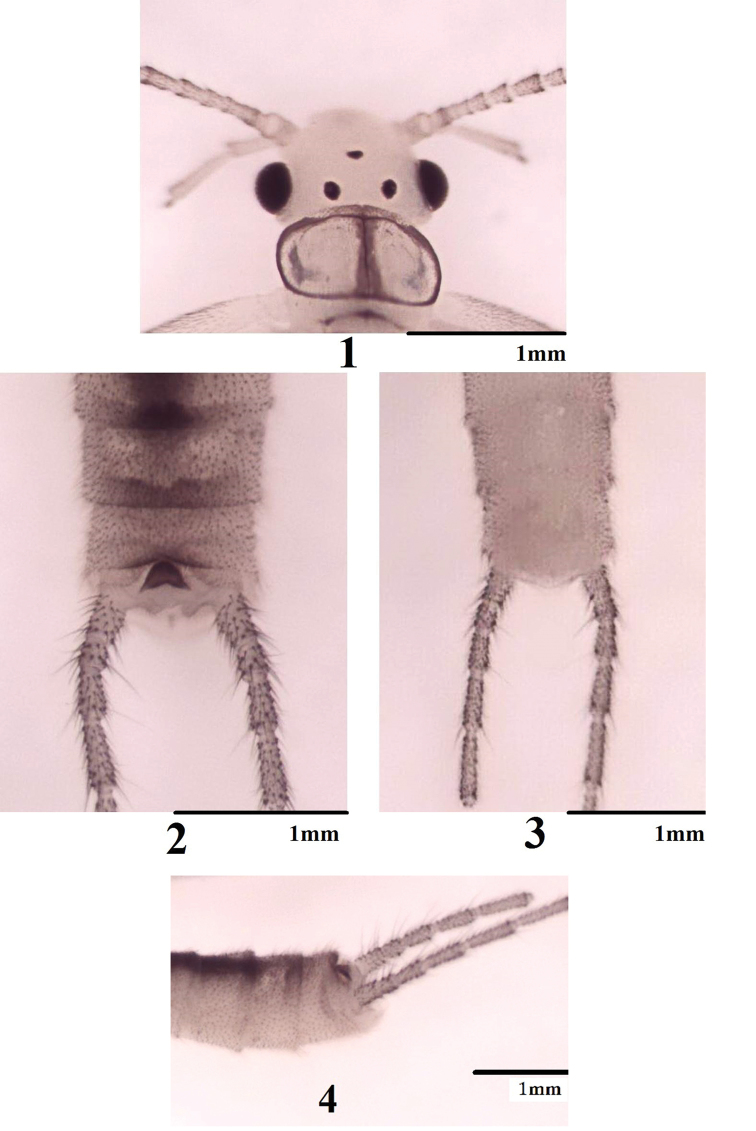
*Haploperla triangulata* sp. n. **1** Head and pronotum, dorsal view **2** Male terminalia, dorsal view **3** Male terminalia, ventral view **4** Male terminalia, lateral view.

Triocellate. General color light yellow patterned with dark brown. Head mostly pale yellow without any markings; compound eyes and ocelli black (Fig. 1). Pronotum hyaline, with median stripe and brown margins; meso- and metanota with dark brown W-shaped markings. Wings hyaline; Rs of both wings branched; A_3_ of forewing fused with A_2_ near base; anal field of hindwing small and folded with three veins (Fig. 8). Coxae, tibiae and femura pale, tarsi brown. Abdominal terga 1–8 with a medial wide stripe. Cerci yellowish-brown with long brown setae (Figs 9, 10).

**Figures 5–8.**
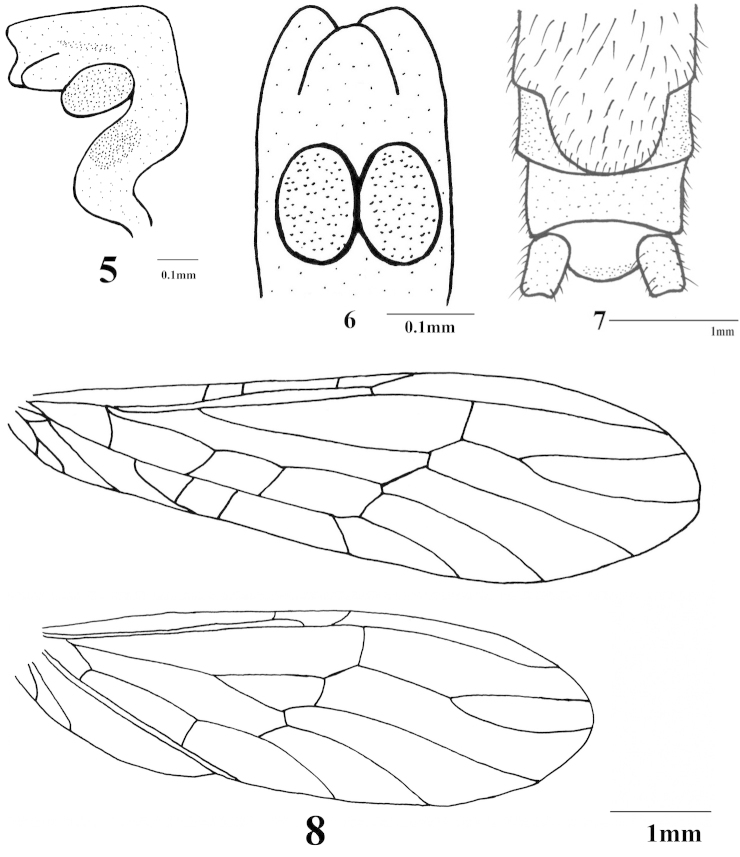
*Haploperla triangulata* sp. n. **5** Aedeagus, lateral view **6** Aedeagus, ventroapical view **7** Female subgenital plate, ventral view **8** Wings.

**Figures 9–10.**
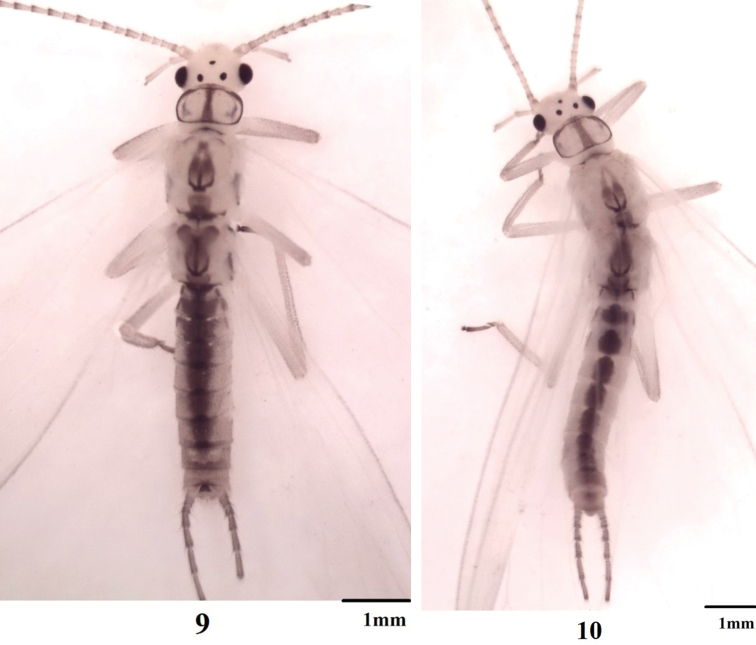
*Haploperla triangulata*, sp. n. **9** Male habitus, dorsal view **10** Female habitus, dorsal view.

#### Male.

Forewing length 6.5–7.0 mm, hindwing length 5.5–6.0 mm. Posterior margin of tergum 9 dark and slightly concave, covered with fine hairs. Tergum 10 divided (Fig. 2). Subgenital plate arising from tergum 9 to tip of the abdomen, slightly tapering at tip (Fig. 3). Subanal process simple, pale and subtriangular in shape. Epiproct mostly sclerotized and hairless, sub-triangular in dorsal view with a blunt tip (Figs 2, 4). Aedeagus membranous and curved ventrally, ventrally with two median situated elliptical lobes, subapically with a plump lobe (Figs 5, 6).

#### Female.

Forewing length 7.0–7.5 mm, hindwing length 6.0–6.5 mm. General pattern similar to males. Abdominal segments 1–8 with median brown strip (Fig. 10). Ventral surface and terminalia without markings. Subgenital plate distinct, originating from tergum 8 to the posterior margin of tergum 9; posterior margin slightly protruding, forming a blunt lobe (Fig. 7).

#### Etymology.

The species epithet refers to the sub-triangular shape of the epiproct.

#### Distribution.

China (Qinghai province).

#### Remarks.

We describe a new species of the genus *Haploperla*, thereby increasing the total number of *Haploperla* species recorded in China to five. More *Haploperla* species are expected to be found in China in the future because the geographical conditions ensure suitable resources for stoneflies. More studies are needed to enrich our understanding of *Haploperla*.

## Supplementary Material

XML Treatment for Haploperla triangulata
